# Supplementary material for: Methyl-Jasmonate Functions as a Molecular Switch Promoting Cross-Talk between Pathways for the Biosynthesis of Isoprenoid Backbones Used to Modify Proteins in Plants
Source: Plants (Basel). 2024 Apr 16;13(8):1110. doi: 10.3390/plants13081110 (PMC11055089; doi:10.3390/plants13081110)
Supplement: Supplementary file 1 [file plants-13-01110-s001.zip › plants-2839341-supplementary.pdf]

	CONTROL			SCREEN
	FOS-treated GFP-CaM-CVIL line	Treatment	Concentrations	Reversion assay on FOS-treated GFP-CaM-CVIL line
	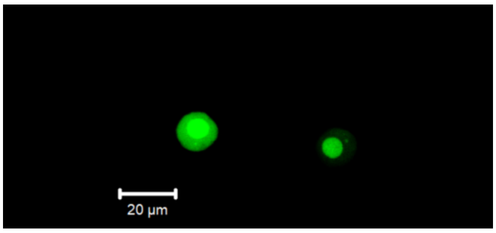	**FOS + MeJA**	100 µM/30 µM	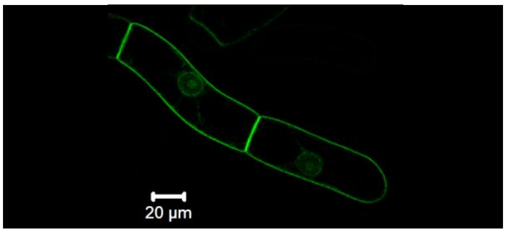
	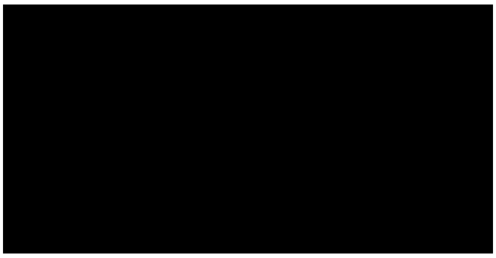	**FOS + ABA**	100 µM/10 µM	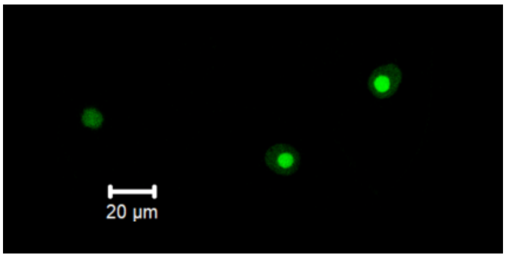
	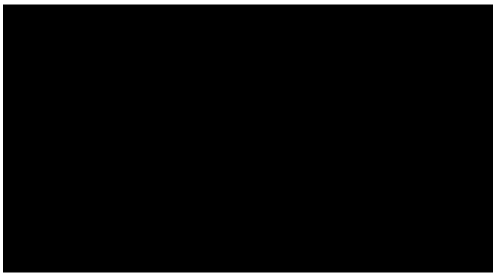	**FOS + IAA**	100 µM/10 µM	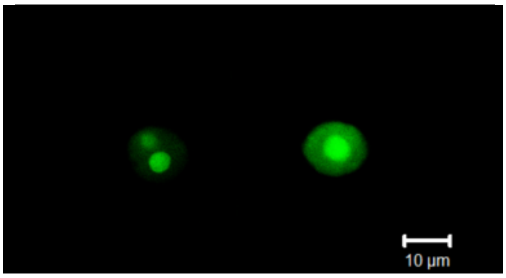
	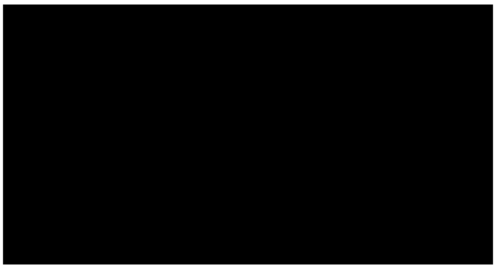	**FOS + NPA**	100 µM/5 µM	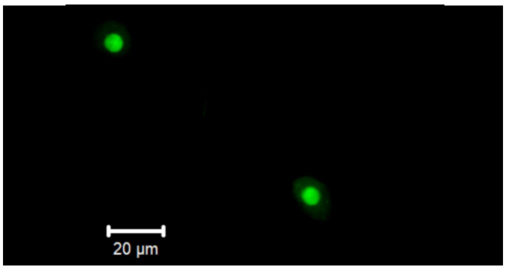
	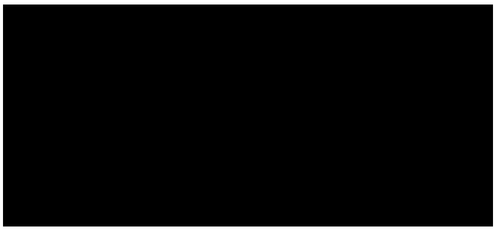	**FOS + kinetin**	100 µM/10 µM	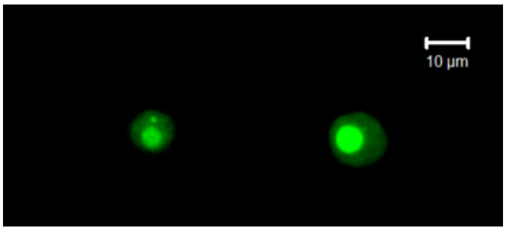
	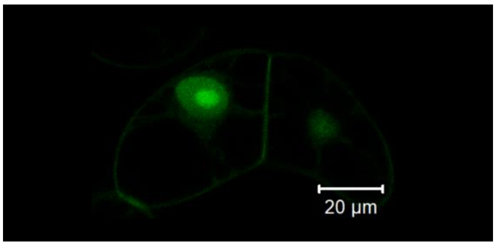	**FOS + MeJA**	50 µM/20 µM	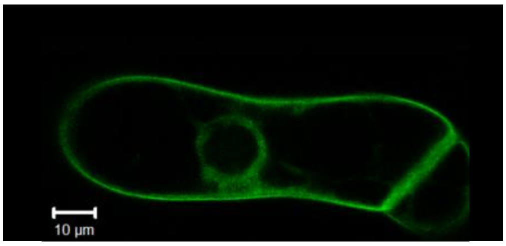
	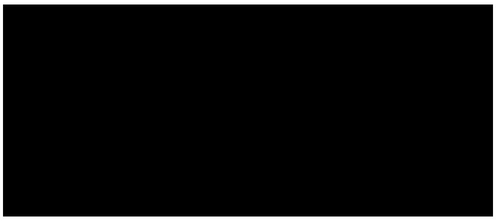	**FOS + Ethephon**	50 µM/100 µM	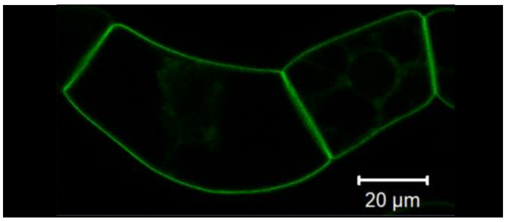

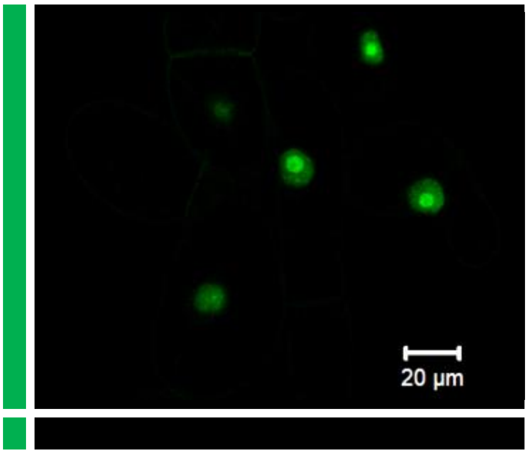

**FOS + pectocellulosic enzyme mix**	100 µM/10 µL

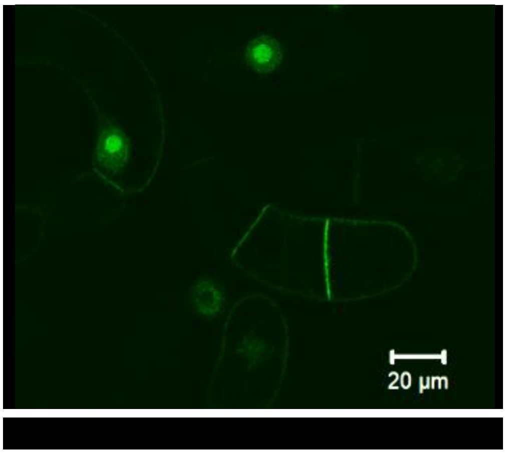

Figure S1. A selection of images gathered by assessing conditions that restore the fluorescence localization of GFP-CaM-CVIL in the plasma membrane in the presence of fosmidomycin (FOS). The pectocellulosic enzyme mix contained 3% Cellulase R10 (Yakult Honsha Co., Ltd, Japan), 0.2% Macerozyme (Yakult Honsha Co., Ltd, Japan), 0.1% Pectolyase (Yakult Honsha Co., Ltd, Japan). N-1-naphthylphthalamic acid (NPA), is a polar auxin transport inhibitor. ABA: abscisic acid; IAA: indole-3-acetic acid; MeJA: jasmonic acid methyl esther. The color on the left side indicates that pictures were taken on the same day and that the same batch of cells has been treated.

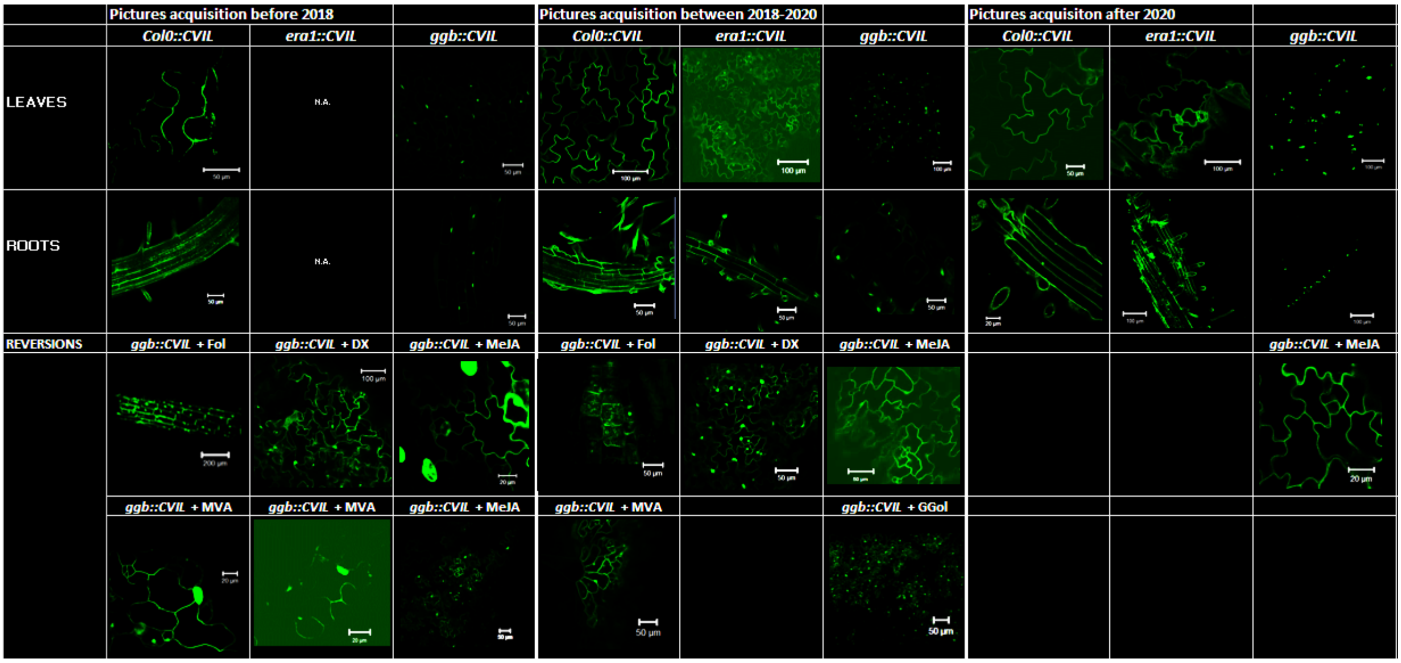

Figure S2. Expression and subcellular localization of GFP-CaM-CVIL proteins in Arabidopsis KO mutant lines over time. Note that over time, the *ggb* mutant remains the only mutant unable to prenylate the GFP-CaM-CVIL protein (nuclear localization). Reversions with farnesol (Fol), mevalonic acid (MVA) and MeJA compared to geranylgeraniol (GGol) and deoxy-D-xylulose (DX) are illustrated. N.A. not available.

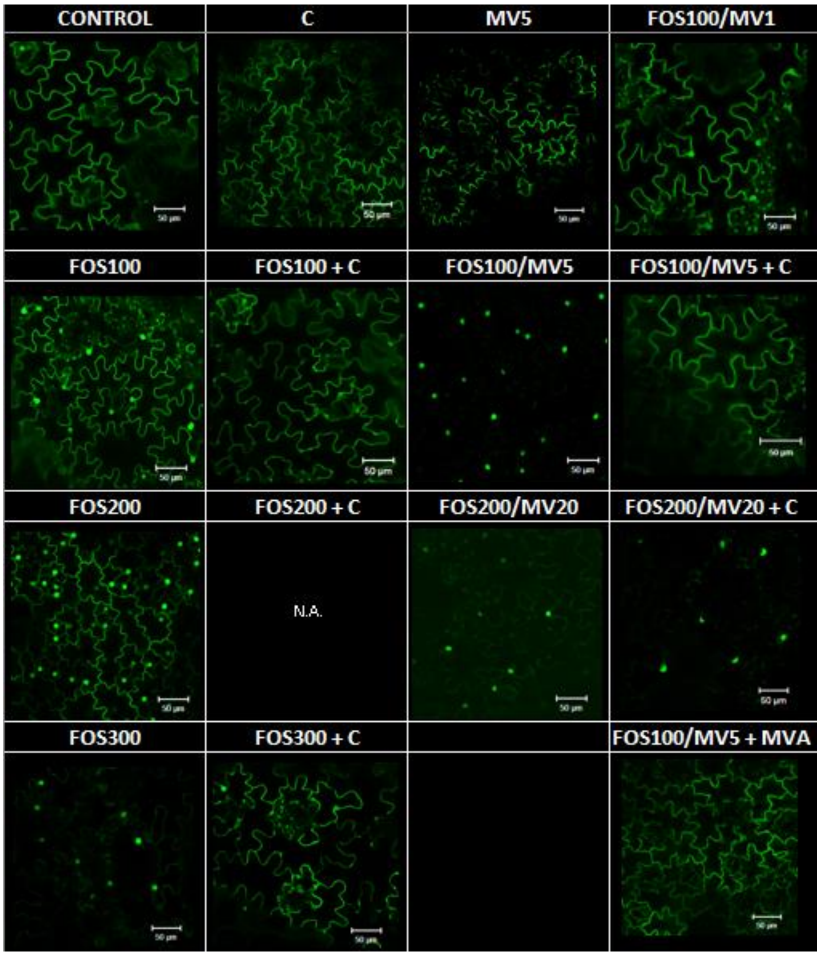

Figure S3. Determination of the concentrations of fosmidomycin (FOS) and mevinolin (MV) to be used to assess the ability of cellulase (C) to overcome prenylation inhibition in *Nicotiana tabacum* leaf disks. Fluorescent nuclei correspond to non-prenylated GFP-CaM-CVIL proteins, while membrane localization in tobacco epidermal cells corresponds to prenylated forms. FOS100 in combination of MV5 is sufficient to block completely protein prenylation (only the nuclei are labelled) and allows reversion with 0.5% of cellulase (FOS100/MV5 + C) illustrated by membrane localization alone. N.A. not available. The white bar represents 50 µm.

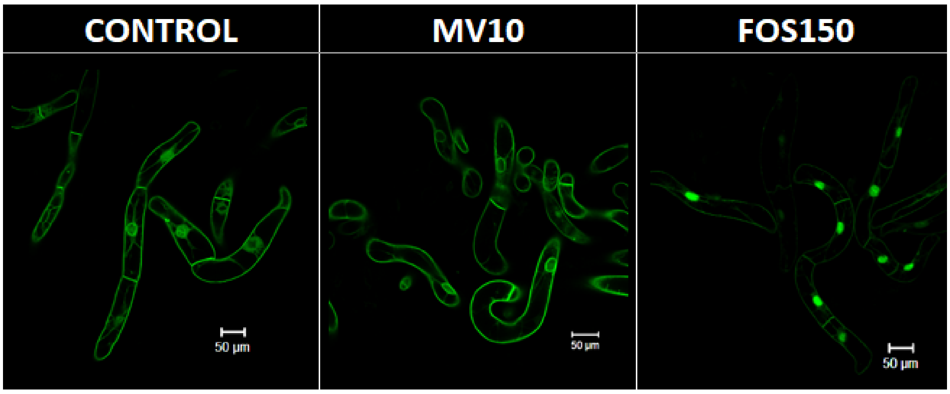

Figure S4. Mevinolin cannot delocalize the fluorescence of GFP-CaM-CVIL from the membrane into the nucleus. Tobacco BY-2 cells were treated for 3 hours with MV or fosmidomycin, before GFP-CaM-CVIL was induced by addition of 30 µM of dexamethasone.
